# Development of novel polymer haemoglobin based particles as an antioxidant, antibacterial and an oxygen carrier agents

**DOI:** 10.1038/s41598-024-53548-5

**Published:** 2024-02-06

**Authors:** Muhammad Abdul Majid, Hafeez Ullah, Ali Mohammad Alshehri, Rukhsana Tabassum, Abdul Aleem, Asad ur Rehman Khan, Zahida Batool, Aalia Nazir, Ismat Bibi

**Affiliations:** 1 Biophotonics Imaging Techniques Laboratory, Institute of Physics, The Islamia University of Bahawalpur, Bahawalpur, Pakistan; 2 Department of Physics, King Khalid University, P.O. Box 9004, 61413 Abha, Saudi Arabia; 3 Institute of Chemistry, The Islamia University of Bahawalpur, Bahawalpur, Pakistan

**Keywords:** Biophysics, Health care

## Abstract

This innovative work aims to develop highly biocompatible and degradable nanoparticles by encapsulating haemoglobin (Hb) within poly-ε-caprolactone for novel biomedical applications. We used a modified double emulsion solvent evaporation method to fabricate the particles. A Scanning electron microscope (SEM) characterized them for surface morphology. Fourier Transform Infrared Spectroscopy (FTIR) and Ultraviolet–visible spectroscopies (UV–visible) elucidated preserved chemical and biological structure of encapsulated haemoglobin. The airproof equilibrium apparatus obtained the oxygen-carrying capacity and P_50_ values. The DPPH assay assessed free radical scavenging potential. The antibacterial properties were observed using four different bacterial strains by disk diffusion method. The MTT assay investigates the cytotoxic effects on mouse fibroblast cultured cell lines (L-929). The MTT assay showed that nanoparticles have no toxicity over large concentrations. The well-preserved structure of Hb within particles, no toxicity, high oxygen affinity, P_50_ value, and IC50 values open the area of new research, which may be used as artificial oxygen carriers, antioxidant, and antibacterial agents, potential therapeutic agents as well as drug carrier particles to treat the cancerous cells. The novelty of this work is the antioxidant and antibacterial properties of developed nanoparticles are not been reported yet. Results showed that the prepared particles have strong antioxidant and antibacterial potential.

## Introduction

In recent research, bio-polymers have garnered significant interest due to their low toxicity, high biocompatibility, and biodegradability within human body tissues or cells. These biopolymers exhibit great potential in various biomedical applications, serving as artificial oxygen carriers, antioxidants, antibacterial agents, contrast agents in diagnostics, and drug delivery agents in cancer treatments. Numerous studies have reported on the applications of both natural and artificial polymers, including PCL, PLA (Polylactic acid), PEG (polyethylene glycol), Poly-glycolic acid (PGA), PLGA (Poly lactic-co-glycolic-acid), and PTMC (Poly tri-methylene carbonate), in various medical contexts^1–3^.

From the main polymers, poly-caprolactone is a semi-crystalline at room and human body temperatures. Its melting temperature Tm is approximately 60 °C^4^. Albertsson et al. previously reported that the crystalline structure was obtained from monomers of ε-caprolactone by ring-opening polymerization followed by different non-hazardous polymerization mechanisms^5^. PCL possesses a unique combination of properties that distinguish it from other polymers, rendering it advantageous for numerous biomedical applications. Here, some significant properties are listed, highlighting PCL as a suitable material for biomedical applications: the biodegradability of PCL makes it an ideal polymer for temporary medical implants designed to degrade and be absorbed by the body over time^6^. The biocompatibility of PCL makes it a good choice for medical devices or implants. A low melting point allows PCL to be moulded or shaped into various forms, making it useful for many applications as expressed by Khan et al.^7^. Toughness and flexibility make it a good choice for applications that require durable materials. The versatility of PCL is to combine it with other materials to achieve particular properties, making it an adaptable polymer with an extensive range of potential uses^6,8^. The degradation parameter is so relative for biopolymers while using them in the medical field. The PCL has a long degradation time due to less repeated ester linkages per monomer. The degradation time of PCL in any biological medium was about 2–3 years^9^. In tissue engineering, PCL scaffolds are utilized with cells to stimulate tissue redevelopment. Drug delivery represents another facet of PCL's medical applications, where it serves as a carrier for drugs and therapeutic agents encapsulated in nanoparticles. The sustained release of doses over time positions PCL as an effective and sustainable drug delivery agent. Moreover, the use of PCL in wound healing treatments has the potential to promote healing and prevent infection^10,11^. Due to its biodegradability, biocompatibility, and relatively cheaper to produce in comparison with other polymers, the FDA approved the PCL for many clinical and medical applications^12^. So PCL got the keen interest of researchers in many biomedical applications reported by Bikiaris et al. such as drug carriers, pharmaceutics, tissue engineering, artificial cells, and artificial oxygen carriers^13^. The present study showed the usage of PCL and haemoglobin to make PCL-Hb-based nanoparticles for various biomedical applications.

Haemoglobin (Hb) is a protein and part of red blood cells^14^. The function of haemoglobin is to transport oxygen from the lungs to the individual tissues of the body^15^. Nowadays, the need for artificial blood (oxygen carriers) increased so much due to the non-availability of donors and the increased rate of blood-related diseases^16^. The blood transfusion service is encountering difficulties in the demand for safe and promptly available blood products. Williamson et al. explored so many challenges like limited shelf-life of blood products, blood group incompatibility, the potential for transfusion-transmitted infections like HIV, and inadequate supply of blood were faced^17^. The global trend towards an increased demand for blood products in medical treatments, including aging-related diseases, cancers, and other conditions, has further widened the gap between supply and demand. Consequently, there is a growing interest in the development of blood substitutes. These substitutes may be employed as artificial oxygen carriers, Hb-based oxygen carriers, and stem cell-derived red blood cells. However, further research and development are necessary to ensure that these alternatives are safe, effective, and widely available for those who require them^18,19^. Anomalies in the structure or function of haemoglobin can give rise to severe illnesses like thalassemia, sickle cell disease, or an abnormal quantity of haemoglobin, which includes conditions like iron deficiency anaemia^20^. Additionally, if there is a lack of proper regulation of intracellular 2,3-diphosphoglycerate in free haemoglobin, it leads to an increased affinity for oxygen. This change makes it ineffective in efficiently supplying oxygen to the tissues^20,21^.

Luckily, researchers discovered that chemically modifying or physically encapsulating Hb proves to be a successful strategy for stabilizing its structure while preserving its oxygen-carrying capacity^22^. The encapsulation process utilizing various biopolymers allows for the alteration of various reactive sites of Hb. Consequently, Hb undergoes modification or encapsulation using biodegradable polymeric substances. These materials offer excellent design flexibility, favorable biocompatibility, and the ability to provide biological shielding. This broadens the potential biomedical applications of biomaterials associated with Hb^23,24^. Utilizing the distinctive physiological functions and physicochemical characteristics of Hb, biomaterials linked to Hb, including haemoglobin-based oxygen carriers (HBOCs), exhibit promising and extensive potential for use as substitutes for blood. Instances where blood transfusions are urgently required, like in cases of severe anaemia, traumatic injuries, and preoperative readiness, amplify the necessity for a greater supply of blood resources^25^. Researchers have revealed that providing oxygen through biomaterials connected to hemoglobin mitigates hypoxia within the tumor microenvironment (TME). This mitigation suppresses tumor growth and, concurrently, enhances the production of reactive oxygen species (ROS), prompting apoptosis or necrosis in cancer cells. This effect will be further amplified when combined with radiotherapy (RT), photodynamic therapy (PDT), or sonodynamic therapy (SDT)^26^.

Furthermore, haemoglobin (Hb) incorporated into biomaterials can initiate the Fenton reaction and create hydroxyl radicals (•OH). Such radicals possess the ability to eliminate tumor cells^27^ as well as bacteria^28^, particularly in postoperative infected wounds^27^. Ibrahim et al. reported that bovine haemoglobin was the source of peptides with biological activities^29^. In a study reported by Parish et al. the cyanogen bromide hydrolysed human haemoglobin to produce four antimicrobial fragments that have inhibitory resistance against gram-positive and gram-negative bacteria^30^. During peptic digestion of bovine haemoglobin at a low degree of hydrolysis, Nedjar-Arroume et al. identified and characterized 24 novel and remarkable antibacterial peptides^31^. Many studies reported the usage of haemoglobin with other materials in the development of several active antibacterial agents^32–34^.

Additionally, biomaterials associated with Hb can transport various gases beyond oxygen, such as carbon monoxide (CO) and nitric oxide (NO), contributing to the enhancement of anti-inflammatory processes^35^. In summary, due to the swift advancements in science and technology, we have the confidence to develop modified multipurpose Hb-related biomaterials to overcome the current limitations. This progress will eventually lead to their practical use in clinical settings, playing a significant role in the betterment of human health in various medical applications^36^. For this purpose, hemoglobin has been utilized with a matrix polymer (PCL) obtained from human or bovine sources to generate artificial oxygen carriers and drug delivery agents, along with various applications such as antioxidants and antibacterial agents. Hemoglobin is encapsulated with PCL to form nanoparticles with the required size, shape, oxygen-carrying capacity, and porous channel properties. Several methods are employed to generate PCL-Hb-based micro or nanoparticles with suitable properties, including single emulsion, double emulsion, solvent evaporation, layer-by-layer, electro-hydrodynamic jetting, and electro-spinning^37,38^.

In this study, the nanoparticles were synthesized with a modified double emulsion solvent evaporation method using PCL as matrix polymer and Hb as an encapsulating agent. The double emulsion method is beneficial for particle preparation because it helps to control the particle’s size and their encapsulation efficiency using different solvents and polyvinyl alcohol (PVA) as an emulsifier. This study explored the properties aside from its low-cost nature, PCL is also able to be modified to meet the needs of structure, biocompatibility, encapsulation properties, and large surfaces for interacting with biomolecules, making it suitable for our study since our study explored new aspects and applications. The sole purpose of this study is to report the unexplored antioxidant potential for the first time and in vitro antibacterial analysis using polymer-encapsulated haemoglobin. Similarly, investigate the toxicity of the particles to verify their biocompatibility. Furthermore, the oxygen-carrying ability and P_50_ values were measured to compare the properties and functioning with native bovine-Hb to reconnoitre their numerous applications in the biomedical field.

## Material and methods

### Materials

Main bio-polymer PCL (Poly-ε-caprolactone) of MW 45 k, bovine-Hb in lyophilized form (Hydrophobic Nature), ethyl acetate (EA)/dichloromethane (DCM) of (1%w/w) used as an organic solvent and polyvinyl alcohol (PVA) used as an emulsifying agent, were purchased from Sigma Aldrich. We acquired the bacterial strains (2-g positive and 2-g negative) from the pathology department at Bahawal Victoria Hospital, Bahawalpur. We obtained the salts for dialysate solution from the chemical laboratory of The Islamia University of Bahawalpur, Pakistan. Phosphate buffer solution (PBS of 10 mg/mL having PH ≅ 7.4) used disodium hydrogen phosphate (2.38 g), potassium dihydrogen phosphate (0.19 g), and sodium chloride in water to make ~ 1000 mL to acquire the suitable PH. We obtained the mouse fibroblastic adherent cell line (L-929) from the Department of Animal Husbandry, Punjab, Pakistan.

### Methods

#### Synthesis of nano-particles

**Figure 1:**
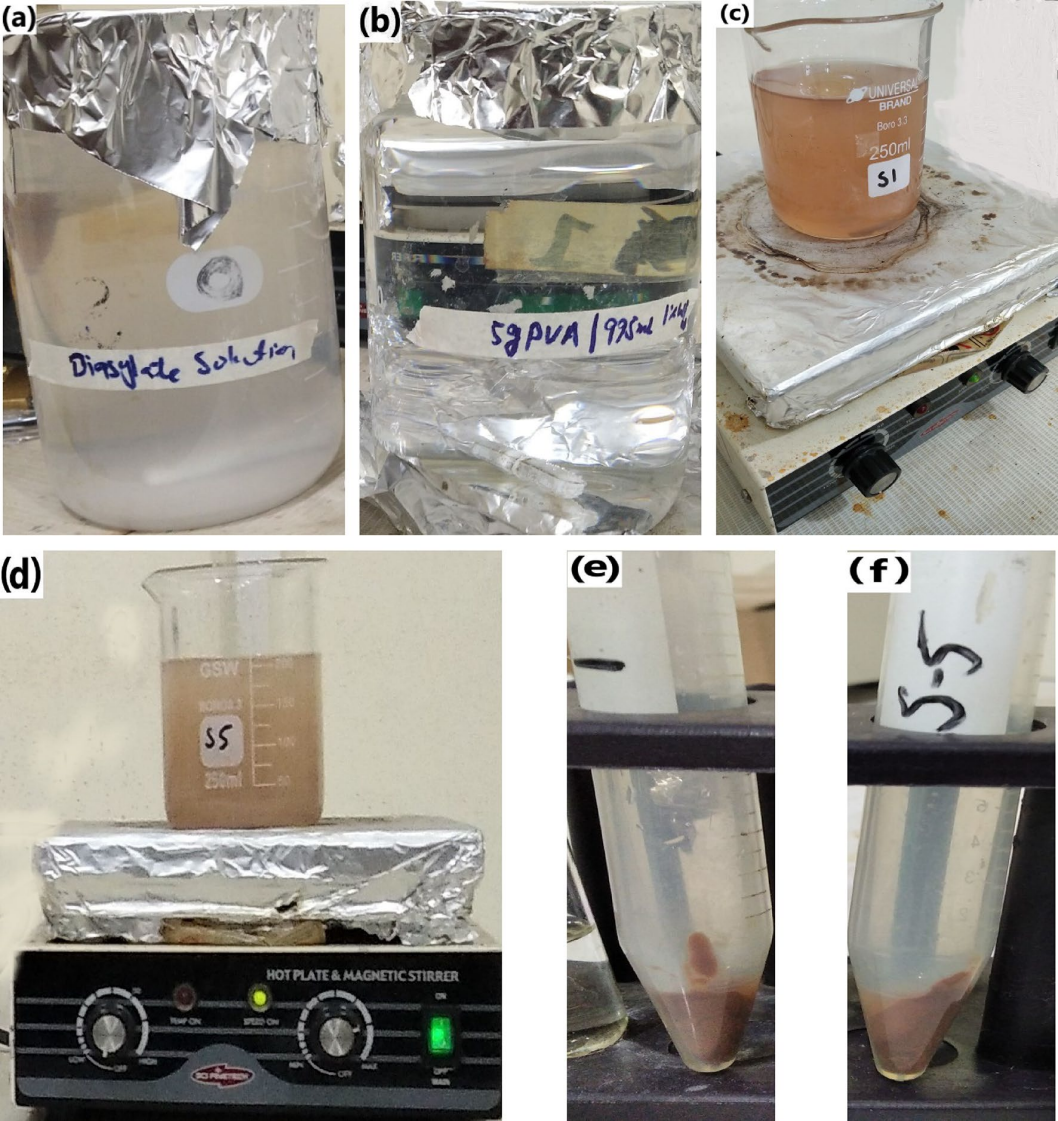
Methodology; (**a**) Dialysate solution to dissolve lyophilised haemoglobin, (**b**) PVA as an emulsifying agent, (**c**) Stirring of final emulsion (PCL-Hb_1%PVA_), (**d**) Stirring of final emulsion (PCL-Hb_5%PVA_), and (**e** & **f**) final wet sample (PCL-Hb particles).

The PCL-Hb particles were developed by the revised double-emulsion (w/o/w) solvent evaporation method, previously explained by Rebecca, Malikmammodov and Freytag et al.^1,2,38^. Firstly, 10 mg of biopolymer (PCL) was added to a 13 mm * 100 mm test tube. Subsequently, 5 ml of 1% (w/w) Ethyl acetate/Dichloromethane/Acetone, serving as an organic solvent, was transferred to the polymer sample using a serological pipette^31^. The polymer was then left to dissolve overnight. The following day, if the solvent had evaporated, additional solvent would be added for further experiments. Initially, bovine hemoglobin (BHb) was in lyophilized form. The buffer solution was prepared using various salts listed in Table 1 to dissolve BHb and achieve a total concentration of 150 mg/mL. Figure 1a illustrates an isotonic dialysate solution of PH (7.1–7.6)^39^. Secondly, 0.5 mL of BHb solution was added to the polymer solution while vortexing until homogenized, resulting in a polymer-BHb suspension. Solutions of the emulsifying agent PVA were prepared with a 1% w/w solution for S1 and a 5% w/w solution for S5, as illustrated in Fig. 1b. The polymer-BHb solution was then added drop-wise to a test tube containing 2 mL of PVA emulsifier, with vortexing for 15 s bursts over 1–3 min until homogenized. This homogenized solution was poured into 200 mL of PVA solution and stirred for 4 h at 360 rpm, as shown in Fig. 1(c-S1 & d-S5), to vaporize the solvent under standard temperature and pressure (STP) conditions (room temperature ≅ 37 °C and atmospheric pressure). The resulting wet suspension of PCL-Hb nanoparticles underwent centrifugation at 21,000*g for 60 min, yielding nanoparticles of approximately 2–3 mL, as depicted in Fig. 1e and f. Finally, the suspensions were lyophilized and store them at − 80 °C before undergoing further characterizations. Otherwise, the particles could be oven-dried for 1.5 h at 50 °C to obtain solidified PCL-Hb loaded particles. With these steps completed, the samples were ready for further characterization and the required analysis^40^.

**Table 1:** Salts used to prepare dialysate buffer solution.

Salts	NaCl	NaHCO_3_	NaH_2_PO_4_–H_2_O	KCl	CaCl_2_–H_2_O	MgCl_2_–5H_2_O
Weight (g/L)	5.85	3.61	0.44	0.37	0.22	0.08

#### Surface morphology and diameter of particles

The surface morphology and shape of prepared samples was investigated by scanning electron microscopy (JEOL, JSM-6480, and Tokyo, Japan). The samples are coated with gold when prepared on stubs prior to imaging. To evaluate size of PCL-Hb particles the PSD analysis (Particles Size Distribution) has been used for mean diameter calculations.

#### About oxygen carrying capacity

##### FTIR measurements

The FTIR of main polymer-based PCL-Hb loaded composites has been performed with (Thermo Fischer Scientific, Waltham, MA, USA, FTIR) that has been compared with native Hb. The samples are prepared as PCL-Hb particles are washed and dried using deionized water, and then KBr tablets are grind with the sample to make a mixture of transparent tablets for FTIR spectroscopy. The spectra have been observed from 2000 to 1000 cm^−1^^38,41^.

##### Oxygen dissociation curves and P_50_

Oxygen equilibrium saturation curves and P_50_ value are the most represented quantitative analysis to explain the oxygen-carrying capacity of prepared samples comparable to native bovine-Hb. The oxygen equilibrium curve and P_50_ values reveal the best relation between oxygen saturation of O_2_ (%) and oxygen partial pressure of O_2_ (mmHg) for bovine-Hb and PCL-Hb Particles. For the measurements of oxygen dissociation curve and P_50_ values, the method describe by Yu Xiong et al.^42^, with certain modifications. An airproof equilibrium apparatus with a digital oxygen meter named a dissolved oxygen meter (HI93732 N Hanna instruments, Italy) made up of organic glass has been used to detect the partial pressure of dissolved oxygen concerning different partial pressure values^43^. Samples were prepared containing native bovine hemoglobin (Hb) and PCL-Hb particles by re-suspending materials in a phosphate buffer (PBS, 10 mg/mL, pH ≅ 7.4) at a temperature of 37 °C. The procedure involved placing 20 mL of samples into an airtight equilibrium apparatus, where the oxygen pressure was varied from (0–100) mmHg to fully saturate the samples with oxygen (O_2_), measured by an oxygen meter. Then samples are moved to UV–visible near-infrared spectroscopy (classical approach method for ODC measurements) to measure the optical absorbance (A) at 660 nm or 940 nm (UV-2450, Shimadzu, Tokyo, Japan) while absorption at 0 mmHg was known as (A_deoxy_) and at 100 mmHg was known as (A_oxy_) at the same wavelength. To account for evaporation and scattering effects with reference material, the absorbance of bovine Hb was measured at 584 nm, which serves as the isosbestic point for both oxygenated Hb and deoxygenated Hb. The oxygen fractional saturation values at given partial pressure has been calculated by the Eq. (1):

1 $${\text{Oxygen fractional saturation }} = \frac{{A - A_{deoxy} }}{{A_{oxy} - A_{deoxy} }}$$

To de-oxygenate bovine-Hb and respective samples, the sodium dithionite has been added to the solution suspensions. The oxygen fractional saturation (%) was taken on Y-axis and pressure in mmHg was taken on the X-axis to draw the oxygen dissociation curve. Furthermore, the P_50_ value and partial pressure were directly measured from dissociation curves^44,45^.

#### UV–visible spectrum

The UV–visible spectrophotometer (UV-2450, Shimadzu, Tokyo, Japan) was employed to obtain the electronic absorption spectra of PCL-Hb encapsulated particles. For this purpose, the samples were dispersed in dichloromethane/ethyl acetate through sonication for 15 min. The suspension of solvent-dissolved particles was then placed in a cuvette. In the UV–visible spectrophotometer, the solvent was used as a reference in another cuvette. UV–visible electronic absorption spectra indicated the preservation of the primary structure of encapsulated Hb. The samples were exposed to compressed air and N_2_ to observe the reverse binding of oxygen and releasing ability. Electronic absorption spectra were measured using three independent particle samples, ensuring the accuracy of data and sample integrity^46^.

#### Free-radical scavenging efficiency of PCL-Hb loaded particles

The scavenging efficiency was evaluated using the 2,2-diphenyl-1-picrylhydrazyl (DPPH) assay for the PCL-Hb particles intermediated by ethyl acetate/dichloromethane. The antioxidant activity of the particles was assessed at increasing concentrations ranging from 5 to 25 μg/mL. After this, incubate all the samples at 37 °C for 30 min in the dark. The colour transformation has assessed the absorbance using UV–visible spectroscopy at (λ = 517 nm). Ascorbic acid is the reference material used in the experiment. The free radicals (%) scavenging capacities were calculated using the Eq. (2).

2 $${\text{DPPH free}} - {\text{radical scavenging efficiency }}\left( \% \right) \, = \frac{{A_{0} - A_{t} }}{{A_{0} }} \times 100$$

Here ‘A_0_’ represents the absorbance of the control sample (DPPH) and ‘At’ represented the absorbance of the test sample^47^. The activity was performed in triplicates, so the results were an average of three values.

#### Anti-bacterial Activity

The synthesized composites went through antibacterial activity using the recommended disc diffusion method by the National Committee for Clinical Laboratory Standards (NCCLS). Four bacterial strains, 2-g negative (*E.coli* & *Pseudomonas*) and 2-gram-positive (*Bacillus cereus* & *Staphylococcus aureus*), are used with three different concentrations (10 μg/mL, 20 μg/mL, and 30 μg/mL) to perform the said activity. Amican used as standard positive control media. The 38 g of nutrient agar powder (Base Material) was dissolved in 1 L of distilled water to prepare experimental sample agar Petri dishes, followed by heating at 121 °C for 20 min. Then, pour the well-mixed and cooled agar solution into Petri plates (20 mL/plate). The bacterial strains were swabbed to the agar Petri plates with different concentrations and incubated at 37 °C for 24 h^47,48^.

#### In-vitro cell viability (MTT assay)

The toxicity of synthesized particles observed by using 3-(4, 5-dimethylthiazol-2-yl)-2, 5-diphenylterazolium bromide) MTT assay on the mouse fibroblastic cell line (L-929). The DMEM (Dulbecco’s modified Eagle’s medium) with 10% fatal calf serum and 10% antibiotics in a CO_2_-humidified incubator (5% CO_2_/95% air) was used to culture the fibroblast cell lines at 37 °C. The 100 μL of derived cell suspensions with a cell density of (≈12 × 10^4^ cells/well) were distributed into 96-well plates and set overnight (12 h) for incubation to allow cell adherence. After that, treated cells with PCL-Hb particles were exposed to growing concentrations ranging from 50 to 800 μg/mL and set for 48 h to incubate at 37 °C. We used a similar seeded cell line without particles as a control. The MTT assays were well controlled by adding 10 μL MTT (PBS of 10 mg/mL with PH ≅ 7.4) in each well soon after the incubation and again set to incubate for 4 h. Then, remove the culture medium, and the cells were mixed well with 150 μL of the DMSO as solvent^49^. After 10 min of agitation, the formazan dissolved completely, and the absorption was taken at (λ = 570 nm) with the help of a (Spectra Max M5) plate reader. The viability of treated cells was expressed as the percentage of the viability of cells grown in the absence of PCL-Hb particles^50^. On the other hand, the viability of the cells was also measured using Eq. (3).

3 $$Cell \, viability \, \left( \% \right) = \frac{OD(test) - OD(DMSO)}{{OD(control) - OD(DMSO)}} \times 100$$

#### Statistical analysis

The experimental values are assessed as the mean ± standard deviation of two experiments for samples with two/three recaps. We used a student’s t-test to evaluate the experimental data. The P-values were measured (*P* < 0.05) using excel and were reflected significantly for this value.

## Results

### Morphology and average diameter of prepared samples

**Figure 2:**
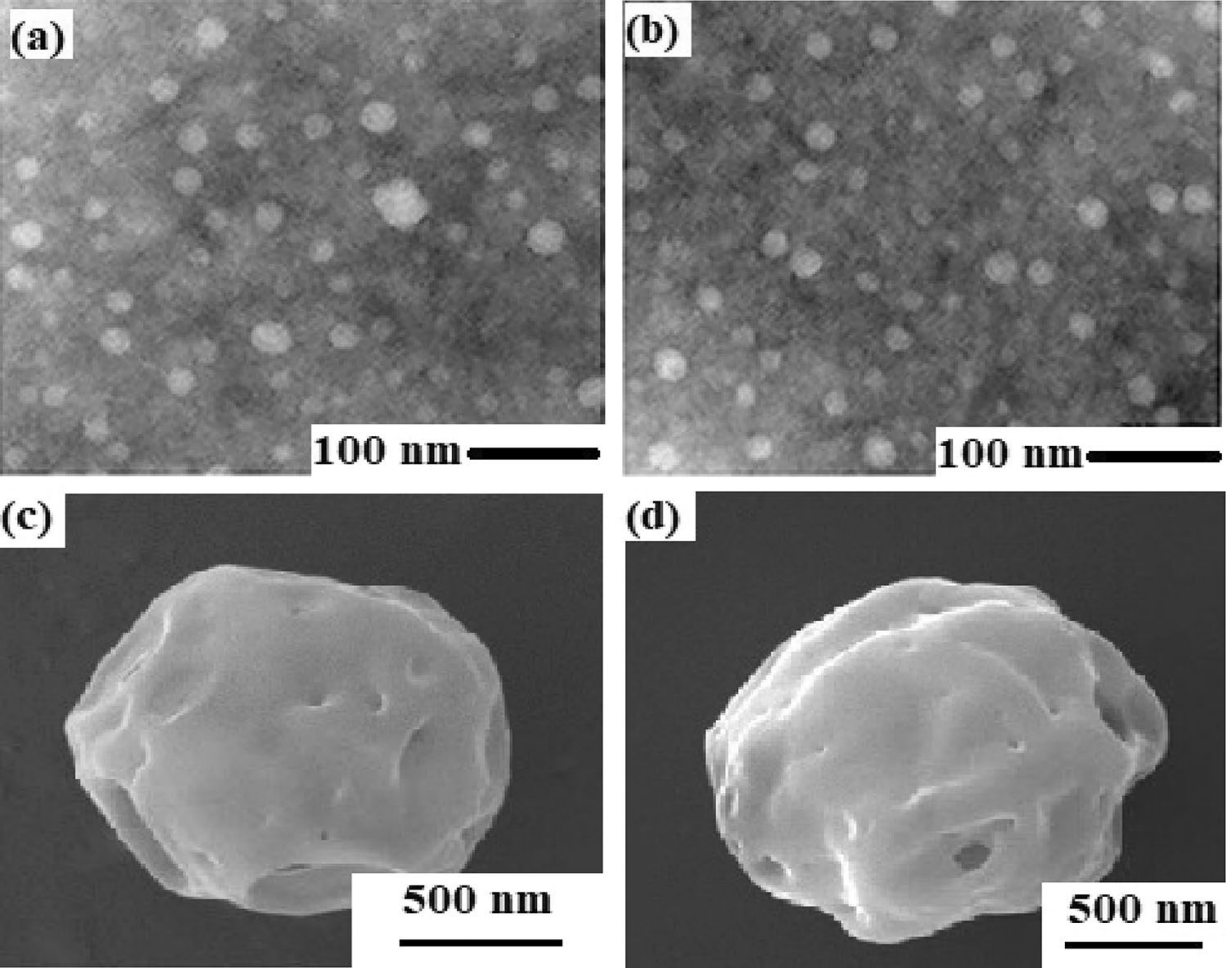
The SEM explored the fairly spherical structure of the developed PCL-HB particles (**a** & **b**). The porous contents are observable on the surface (**c** & **d**).

Morphological analysis of PCL-Hb particles was conducted using scanning electron microscopy. Figure 2a and b illustrates the size, shape, and distribution of particles, which appear homogeneous and fairly spherical. Notably, observable porous contents are present, as shown in Fig. 2c and d, on the surface of the particles. The porous structure of HbP results directly from the solvent diffusion process. It was demonstrated previously by Zhao et al. that the solvent evaporation process efficiently removed the solvent while preserving the chemical structure of Hb. During the solidification procedures, higher temperatures led to the creation of ample pore space. Additionally, the preparation temperature played a significant role in determining the porous structure by influencing the removal rate of solvents^38^.

Principally, the porous structure is devised from solvent diffusion and subsequent solidification processes. This inherent porous structure within HbP is expected to enhance the exchange of oxygen in and out of the material, effectively describing its oxygen-carrying capacity. Particle size distribution (PSD) analysis was used to measure the diameter of the samples, and the average values ranged between 30 to 60 nm. The particle size is highly controlled by re-emulsification and the pre-solidification process of synthesized particles, as strong emulsification results in smaller particles^38,51^.

### Oxygen carrying capacity

The PCL-Hb particles are probable to expedite the oxygen exchange through building porous channel structure of particles. So in this research, the stability of Hb-encapsulated within particles and porous channels evaluation predicts the oxygen carrying capacity.

#### FTIR analysis

It is necessary to maintain the physical and chemical structure of encapsulated Hb in PCL-Hb particles as HbP are expected to provide oxygen in and out through the porous channel. So oxygen carrying capacity is purely related to the modified preparation method and stable/preserved structure of encapsulated Hb.

**Figure 3:**
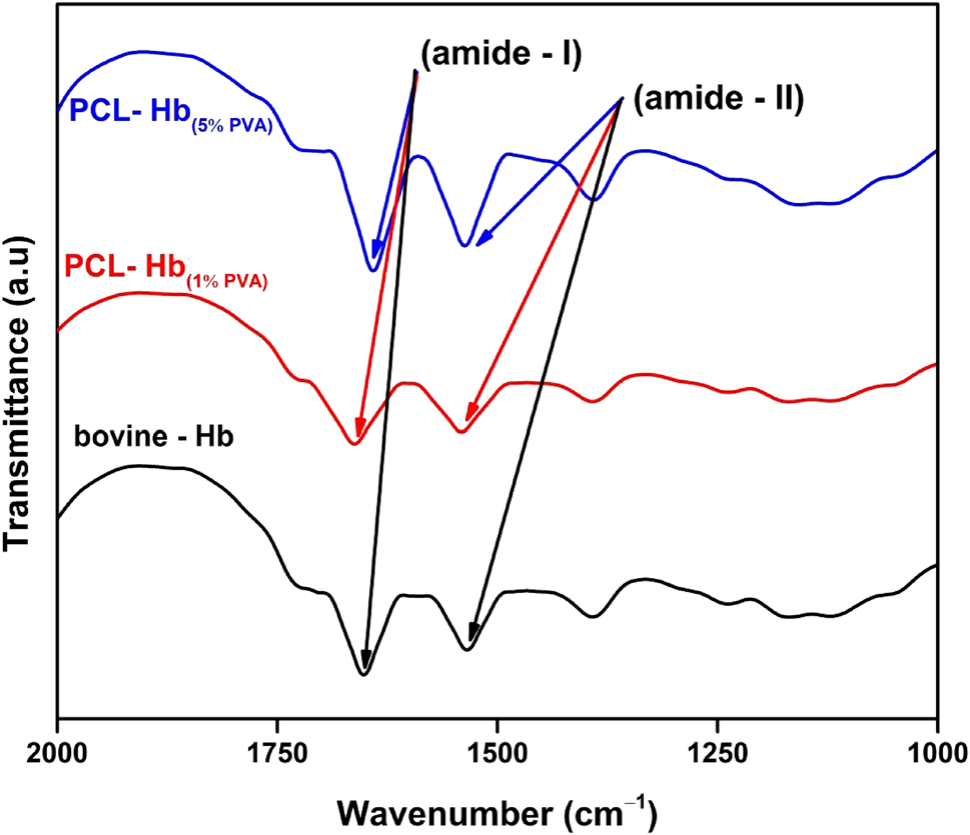
FTIR spectra indicated the identical amide-I and amide-II functional groups of Hb that revealed the preserved chemical and physical structure of particles. Bovine-Hb (Black), PCL-Hb (S1-Red) and PCL-Hb (S5-Blues).

The amide-I band govern the stretching vibration of (C=O) located at 1662 cm^−1^ for S1 (Red) & 1640 cm^−1^ for S5 (Blue), indicate the strong information about the presence of hydrogen bonding and amide-II band originate from in-plan (N–H-bending & C–N-stretching) located at 1540 cm^−1^ for S1 & 1536 cm^−1^ for S5. The spectral features group (amid-I 1650 cm^−1^ & amide-II 1534 cm^−1^) for bovine-Hb as control and the bands of native bovine-Hb and HbP are approximately identical as shown in Fig. 3. These functional features of bovine-Hb are quit identical, as previously reported by Jansman et al. for Hb-based oxygen carriers^41,52^.

#### Oxygen dissociation curve and P_50_ values

**Figure 4:**
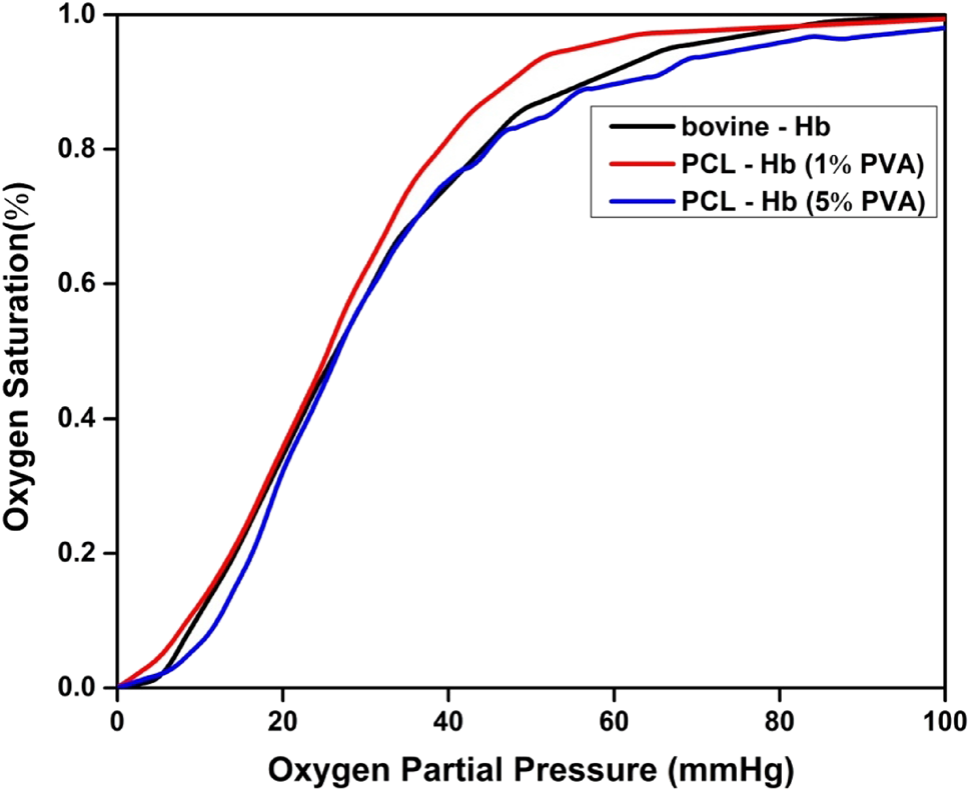
Oxygen dissociation curves of PCL-Hb particles using bovine-Hb as reference. P_50_ values are measured directly form OD curves.

In this study, we aim to verify the efficient oxygen delivery connected with the pore structure, as the encapsulated Hb demonstrated oxygen-carrying capacity results similar to native Hb. The reliability of the calculation method used was also investigated through a literature review, revealing that some polymer compositions exhibit the same oxygen dissociation curve (ODC) profile and P_50_ measurements, as listed in Table 2, comparable to bovine Hb and human Hb as controls. The oxygen-carrying capacity and P_50_ values were expressed through the oxygen dissociation curves, as shown in Fig. 4. The ODC’s of particles are in a similar pattern to native-Hb. The accompanying P_50_ results obtained directly from the ODC curve of S1-red (25.5 mmHg) and S5-blue (26.75 mmHg) are quite a match with P_50_ of native Hb-black (26.5 mmHg), as shown in Fig. 4^53^.

**Table 2:** P50 values of human blood, bovine-Hb and prepared PCL-Hb particles. The activity was performed in triplicates so it is an average of three values (n = 3).

Materials/particles	P_50_ (mmHg)	References
Human blood	27	^54^
PLA Hb-particles	26.5	^53^
Bovine-Hb	26.5	This study
PCL-Hb_1%PVA_ (S1)	25.5	This study
PCL-Hb_5%PVA_ (S5)	26.7	This study

The NIR spectra were used in UV-spectroscopy due to the high penetration of NIR in the polymer matrix for the safe and nondestructive detection of entrapped Hb in polymers. The results of ODC for oxygen saturation with different applied values of oxygen partial pressure revealed similar sigmoidal shapes of ODC curves and P_50_ values with respect to reference materials.

### UV–visible spectrum

The UV–visible spectrophotometer was employed to assess the preservation of the chemical structure, functionality of encapsulated Hb, and an essential property of oxygen transportation in hemoglobin. For this purpose, a solution containing native BHb and particles was prepared and placed in a UV–visible spectrophotometer, utilizing wavelengths in the range of 380–650 nm. Furthermore, absorption peaks, known as the Soret band and Q-band, were observed for both samples, as listed in Table 3.

**Figure 5:**
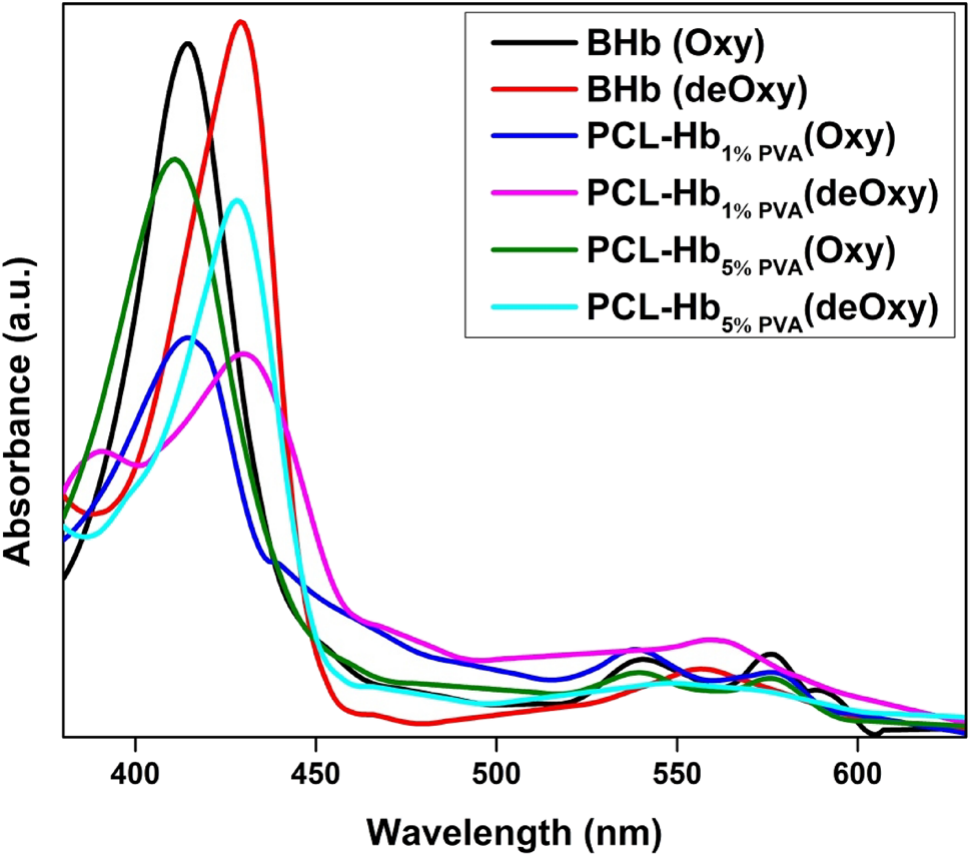
UV–visible spectra of bovine-Hb and PCL-Hb particles at oxygenated and deoxygenated states. Herein BHb represented the bovine-Hb, S1 for PCL-Hb_1%PVA_ and S5 for PCL-Hb_5%PVA_.

The suspension of Hb prepared using a lyophilized form of BHb and the absorption peaks of heme-proteins of oxygenated free BHb suspension are explained in Fig. 5 by a strong band named Soret peak (B-band) that have maximum absorption at ~ 415 nm. Two more peaks (weak band) were also observed around (480–600 nm), known as Q-band. The Q1 & Q2 bands have maximum absorption values at ~ 539 nm and ~ 576 nm, respectively. While purging free BHb with N2 to remove the residual oxygen, the soret peaks show a red shift as maximum absorption was at ~ 430 nm, and a single Q-band was spotted at ~ 556 nm. The peaks observed at ~ 415 nm and ~ 412 nm in the Oxygenated state were called soret peaks. The observed Q-bands for the oxygenated state are shown in Fig. 5. The soret peaks are shifts from previous patterns due to the light scattering effects of particles themself. To achieve the de-oxygenation, 1.5 mg/mL of SDT (sodium dithionite) will be added to prepare the particle suspension as previously reported by Jansman et al.^55^.

**Table 3:** Soret peaks and Q-bands for oxygenated and deoxygenated states of bovine-Hb and PCL-Hb particles.

Materials/Samples	Soret Peaks (nm)		Oxygenated (nm)		Single Q-Band Deoxygenated (nm)	References
	Oxygenated	de-Oxygenated	Q1	Q2		
PCL-Hb_1%PVA_ (S1)	415	431	538	580	560	This study
PCL-Hb_5%PVA_ (S5)	412	430	540	576	547	This study
Free bovine-Hb	415	430	539	576	556	This study
Hb/PDA Np	414	427	542	576	559	^56^
Hb-dNPs	414	430	542	576	554	^57^

### Antioxidant (free radical scavenging activity)

**Figure 6:**
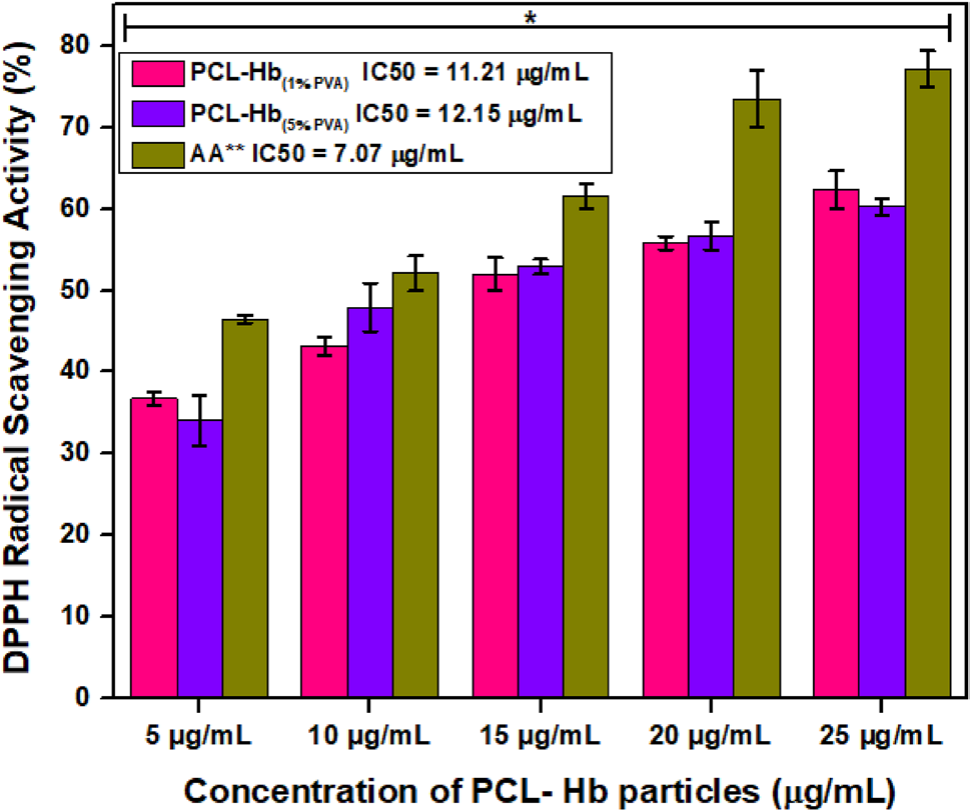
The antioxidant activities of PCL-Hb particles using ascorbic acid as standard by DPPH assay. Experiments were performed in triplicates, so the results were expressed as an average ± SD of three values (n = 3). The error bars represented the deviation for the obtained average value. The quantification has significance of DPPH potential (*for *P* < 0.05) of same samples at increasing concentrations verses standard used.

The DPPH assay is a decolourization method employed to measure the relative antioxidant capacities of materials by assessing their ability to scavenge free radicals. Its excellent reproducibility, stability, and commercial availability make it an easy method to calculate the aforementioned activity^58^. The results obtained were tabulated in Table 4 and graphically presented in Fig. 6 using a standard material. The findings indicate that materials developed with higher concentrations of emulsifiers exhibit heightened antioxidant abilities, as reflected by larger IC50 values. Conversely, particles developed with lower emulsifying agents show reduced antioxidant properties. The higher concentrations of emulsifiers aid in the strong binding of hemoglobin to PCL. This results in the development of stable particles for in-vivo and in-vitro studies as antiradical agents. The measured IC50 value of particles represents the concentration required to inhibit 50% of radicals, with lower IC50 values indicating stronger antioxidant properties. In a study, Hu et al. reported that hemoglobin coated with polymerized poly-dopamine has reduced oxidative properties towards human umbilical vein cells (HUVEC) and can scavenge up to 85% of hydroxyl free radicals^59^. The developed materials can be used as an antioxidant agent to treat the cancerous cells and free radicals produced.

**Table 4:** The IC50 values for PCL-Hb particles and standard used. We performed the activity in triplicates. So, it is an average of three values.

Materials/Samples	% *Scavenging free radical of DPPH at different concentrations (µg/mL)					^⁂^IC50 Values (µg/mL)
	5	10	15	20	25	
PCL-Hb_1%PVA_ (S1)	36.81	43.15	52.05	55.82	62.33	11.21 ± 0.09
PCL-Hb_5%PVA_ (S5)	34.07	47.95	52.91	56.68	60.27	12.15 ± 0.04
AA^⁑^	46.47	52.09	61.52	73.47	77.18	7.07 ± 0.06

% *Scavenging = ((A_o_-A)/A_o_) × 100. Where A_o_ = absorbance of blank, A = absorbance of sample).

AA^**⁑**^ = Ascorbic acid (Standard compound).

IC^**⁂**^_50_ = Concentration of compounds for 50% inhibition of DPPH calculated by non-linear regression.

The PCL-Hb particles have comparable antioxidant measurements to the standard control, as shown in Fig. 6. The minor disparities are mainly due to the encapsulation process, use of solvent concentration, and emulsifying agents, because all these are significantly affecting the basic structure and other properties of PCL-Hb loaded particles.

### Antibacterial analysis

**Figure 7:**
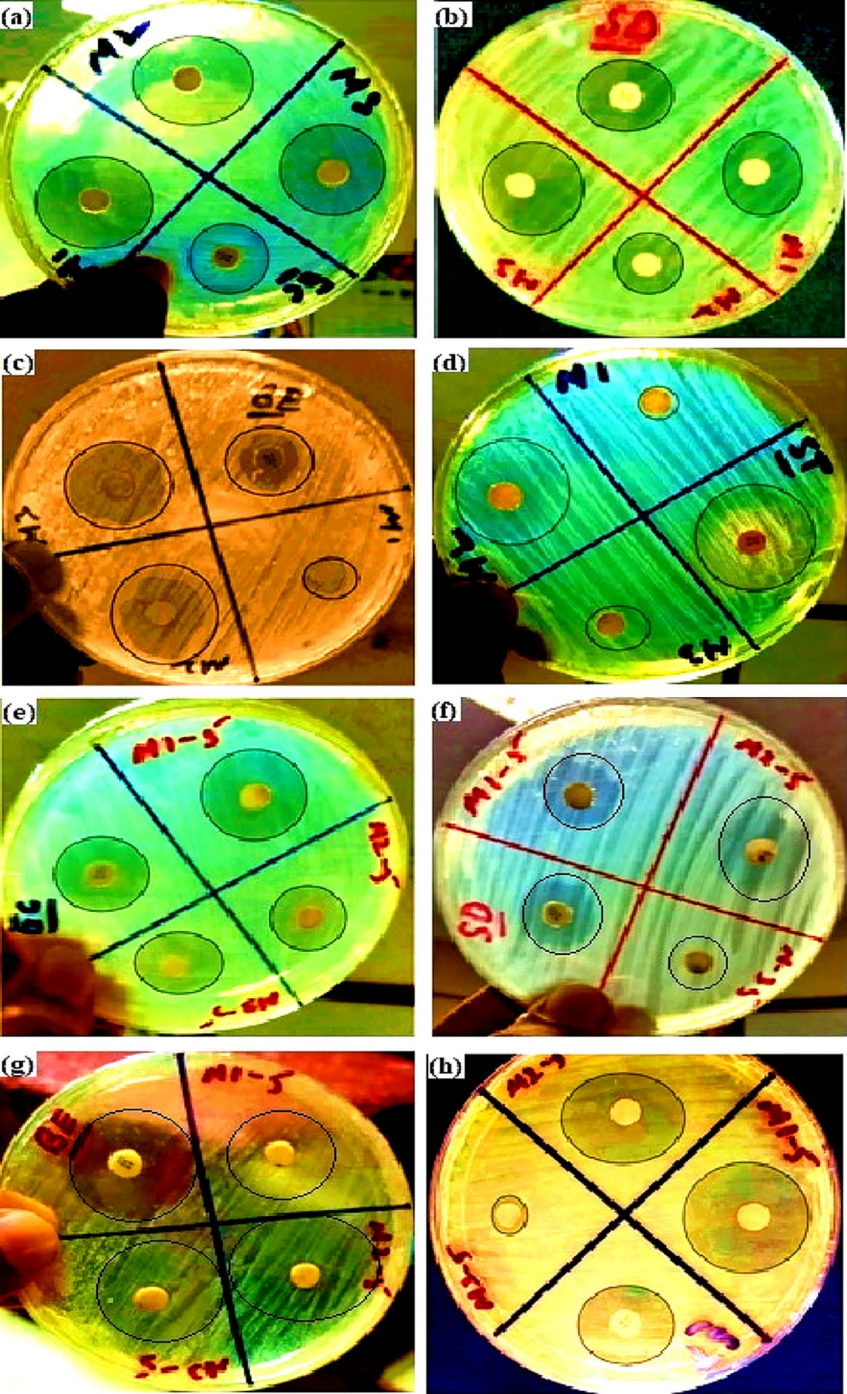
Clear inhibition zones are shown by PCL-Hb particles on different bacterial strains. (**a**) *E.coli*, (**b**) *Pseudomonas*, (**c**) *Bacillus cereus,* and (**d**) *Staphylococcus aureus* had shown the results for PCL-Hb1%PVA (S1) where M1, M2, and M3 represented different particles concentrations on each bacteria. Similarly, (**e**), (**f**), (**g**), and (**h**) have shown the results for PCL-Hb5% PVA (S5) on the same bacterial strains, and concentrations were represented by M1-5, M2-5, and M3-5.

In our work, the polymer-haemoglobin (PCL-Hb) particles are novel and are not reported yet as an antibacterial agent. The PCL and Hb are used individually in different antibacterial properties exploration in composite forms. For this exploration, two-gram negative (*E.coli* & *Pseudomonas*) and two-gram positive (*Bacillus cereus* & *Staphylococcus aureus*) were used to perform the said activity of particles. The remarked values of the zone of inhibition for PCL-Hb particles and standard control media (Amican) are physically presented in Fig. 7a–h and listed in Table 1 (Supporting information file).

**Figure 8:**
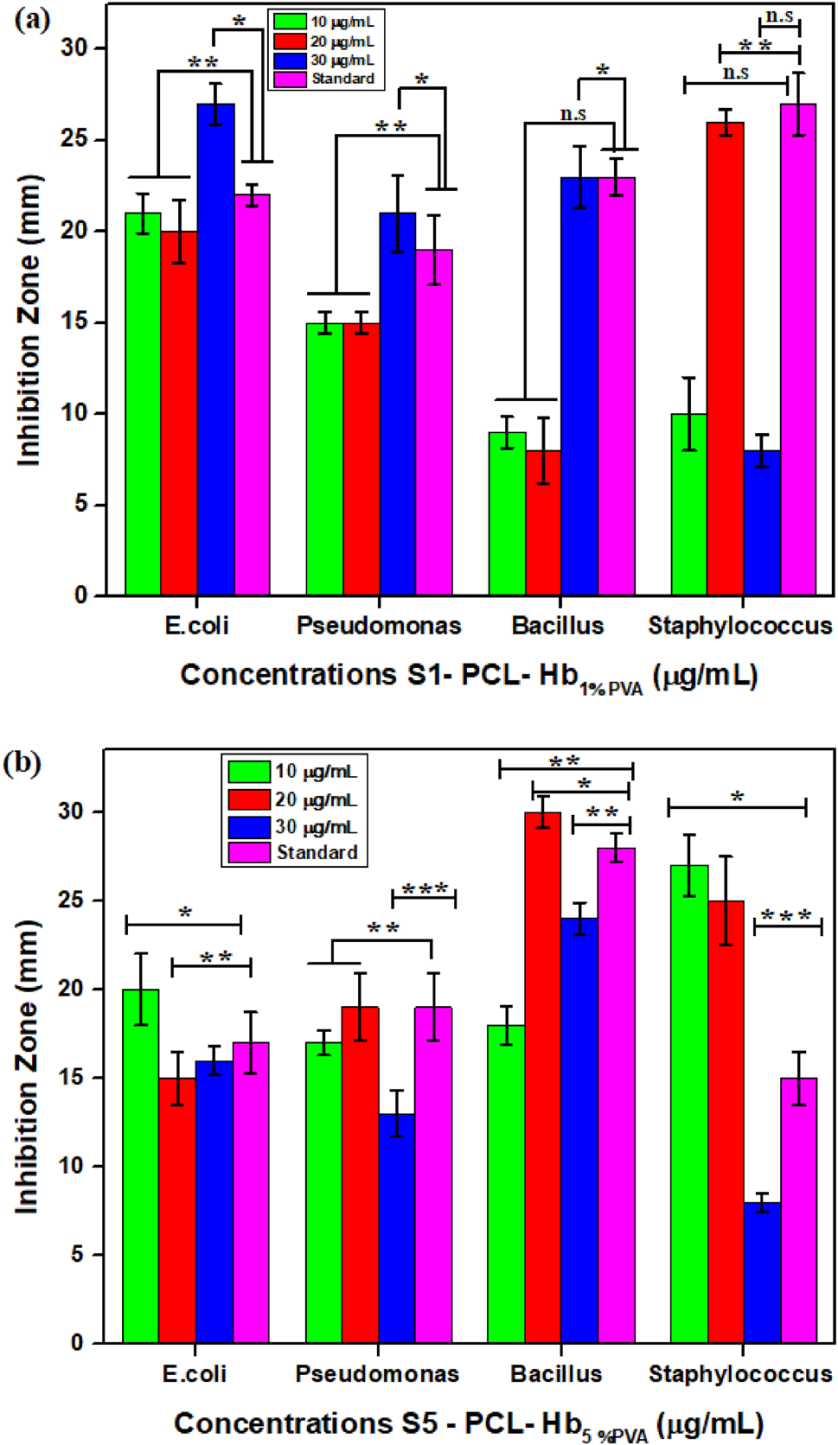
Histogram analysis of particles with two gram-negative & gram-positive bacteria at different concentrations and standard control. (**a**) Zone of inhibitions for PCL-Hb_1%PVA_ (S1) particles. The quantitative analysis showed the effectiveness of same particles verses different bacterial strains at increasing concentrations (**b**) Zone of inhibitions for PCL-Hb_5% PVA_ (S5) particles. The statistical analysis showed that same particles have major impact verses different bacterial strains at increasing concentrations. The error bars expressed the variation for obtaining data that was an average ± SD of three values (n = 3) represented significance difference * for *P* < 0.05, ** for *P* < 0.01, *** for *P* < 0.001, and n.s for non-significant.

The gram-negative strains have similar bacterial growth for both samples as shown in Fig. 7a and e for *E.coli* and *Pseudomonas* as shown in Fig. 7b and f. Results indicated in Fig. 8a and b revealed that the S5 showed higher inhibition zones compared to S1, which may be due to durable emulsification and smaller size. By considering the concentration effect on bacterial activity, S1 has enhanced antibacterial activity over S5 at (30 μg/mL of concentration) on *E.coli*, *Pseudomonas*, and *Bacillus* bacterial strains, as shown in Fig. 8a, while S5 have relatively effective at the concentration of 20 μg/mL, as shown in Fig. 8b. Generally, S1 has effectiveness on gram-negative bacterial strains, and S5 showed efficacy on gram-positive bacterial strains. The inhibition zones were measured in mm and drawn as a histogram. That showed that the ZOI for gram-negative bacteria (*E.coli* & *Pseudomonas*) are relatively small in comparison with gram-positive bacteria (*Bacillus cereus* & *Staphylococcus aureus*) as shown in Fig. 8a and b. The results of said activity explored that the PCL-Hb particles exhibit the antibacterial property and may be used in many medical applications as control media for bacterial growth.

### Cell viability analysis

**Figure 9:**
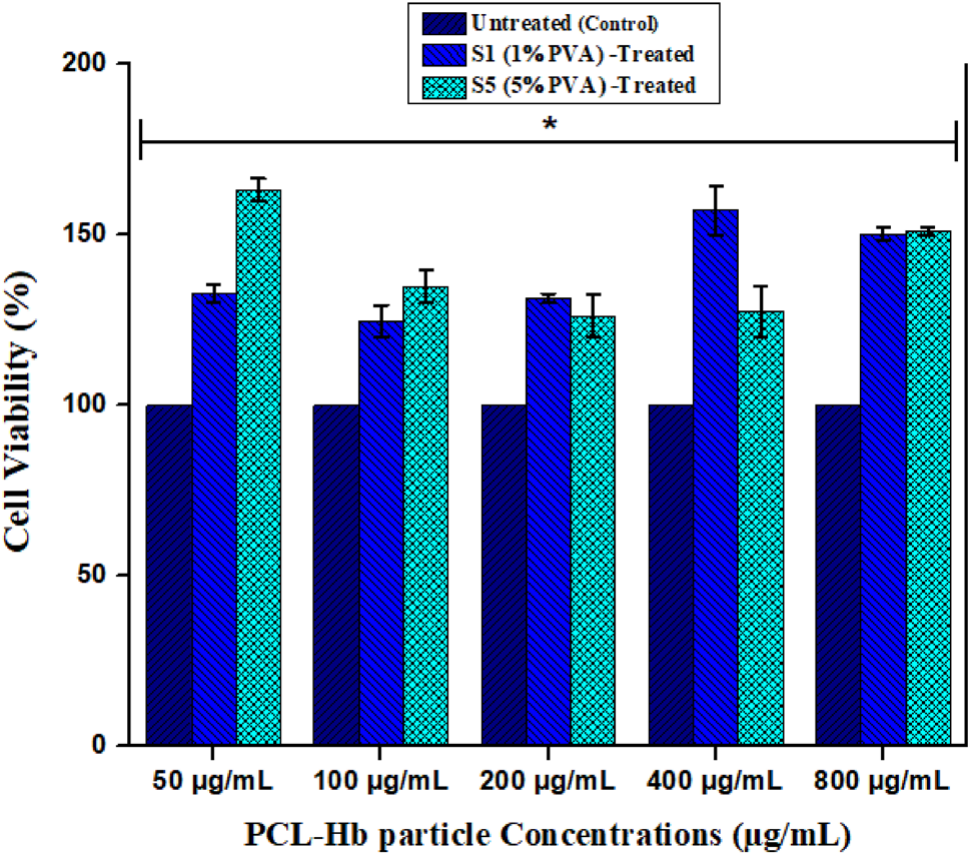
The cell viability analysis using MTT assay using animal fibroblasts cultured cell lines. The error bars represented the deviation for the average of three measurements (n = 3). The statistical data represented the normal growth (Nontoxicity) of cells while having same particles with increasing concentrations verses control media that has significance (*P < 0.05) after 48 h of incubation.

The In-Vitro cell viability was assessed by MTT assay using animal fibroblasts cultured cell line. The outcomes of the said activity are graphically shown in Fig. 9 and listed in Table 5. Results of all cell lines with different concentrations ranging from 50 to 800 μg/mL indicate cell viability after 48 h of incubation compared with untreated (control) cell lines without particles. The concentration of the particle suspensions was determined solely based on the weight of polymers to maintain a consistent dosage of PCL-Hb particles in the cells. The treated animal fibroblast cell lines show usual growth. S1 treated grows slowly in the small concentrations range, and cell viability increases at higher concentrations. Similarly, the cell viability for S5-treated cells is higher at low concentrations and vice versa. So it is observed that the cells proliferated well, and the synthesized particles are nontoxic and cyto-compatible (biocompatible) in large concentration ranges.

**Table 5:** Percentage cell viability of animal fibroblasts cultured cell lines using MTT assay. The results were an average of three measurements as activity was performed in triplicates (n = 3).

Materials/Samples	% Cell Viability of MTT at different concentrations (µg/mL)				
	50	100	200	400	800
PCL-Hb_1%PVA_ (S1)	132.77	124.68	131.38	157.22	150.19
PCL-Hb_5%PVA_ (S5)	163.32	134.95	126.29	127.48	151.06
Untreated (Control)	100				

## Discussion

Biopolymers, being biodegradable, offer significant potential for designing nanoparticles through the encapsulation of hemoglobin using the double emulsion method for various medical applications. The focus is on developing artificial oxygen carriers, antioxidant agents, and antibacterial agents with a stable structure, suitable size and shape, and active functionality of hemoglobin within polymers, which plays a vital role in numerous practical applications. To achieve the required properties, SEM analysis reveals abundant porous contents on the surface of the particles, as shown in Fig. 2c and d. These porous channels aid the particles in carrying oxygen, making them applicable as artificial oxygen carriers. The oxygen-carrying capacity is evaluated by encapsulating Hb within the matrix polymer, as previously verified by Tsapis et al. regarding the drug-carrying ability of polymeric and non-polymeric nanomaterials' porous channel properties^60^. The oxygen-carrying capacity of particles related to the stable structure of encapsulated haemoglobin is justified by FTIR spectroscopy that identifies the protein (Hb) and its functional groups’ response to the IR radiation absorption, which helped to recognize the bimolecular validity of the particles^61^. For the above investigations, the FTIR spectra are observed for native-Hb and encapsulated-Hb, as shown in Fig. 3. The comparison of spectrum indicates the identical amide-I and amide-II groups which means the modified solvent evaporation technique doesn’t alter the encapsulated-Hb structure^62^. Hence they can serve as oxygen carriers artificially^41,52^. Another aspect that verifies the oxygen-carrying capacity is the oxygen dissociation curve and the P_50_ measurements, as shown in Fig. 4. The primary discussion about the oxygen dissociation curve revolves around the observation that a high-pressure value causes Hb to bind with O_2_, forming Oxy-Hb, and reversely releases O_2_ in a low oxygen environment, creating deoxy-Hb. This process has been addressed by Mairbäurl et al. explaining how the valence iron ligands respond to O_2_ binding and unloading correspondingly^63^. The oxygen saturation profile of Oxy-Hb and deoxy-Hb showed different absorption patterns, and by using Eq. (1), the ODC curves are obtained to check the validity of subjected properties. It has been reported previously by Böning et al. that nano-size particles showed longer half-life (circulation time) rather than micro-size particles due to the fast elimination function from blood using a different polymer as the matrix polymer^64^. Micro-size particles have low P_50_ value, but nano-size particles have P_50_ near the bovine-Hb (26.5 mmHg) and human blood (27 mmHg) as well comprises the validity of our prepared nanoparticles functionality^43,64^. The results demonstrated that the PCL-Hb particles have essential properties to carry oxygen. It will allow us to understand how oxygen will be bound and released with Hb in imagination within the lungs and capillaries^65^.

Furthermore, the observed UV–visible spectra ensure the preservation of the chemical structure, functionality, and an elementary property of haemoglobin (Hb) of oxygen transportation. The results of the oxygenated (~ 425 nm) and deoxygenated (Q-bands at ~ 542 nm & ~ 578 nm) property of free Hb to reversely bind and release the oxygen confirmed previously by Chen et al.^56^. Somehow Q-bands disappeared by scattering by particles itself but the soret peaks are well distinguished, which allows seeing the preserved structure and functionality of Hb within PCL-Hb particles^66^. In all cases, slight changes occur in absorption peaks (soret & Q-bands) associated with the existence of protein ligands in Hb as previously well defined by Dessy et al.^67^. The initial soret peak indicated that ferrous ion (Fe^2+^) in Hb was ready to bind the oxygen to form Oxy-Hb. Soon after the binding, the di-oxygen molecule converted into low-spin six coordinated iron porphyrin oxygen. We observe that soret peaks were shifted after the de-oxygenation occurs^68,69^. Previous reports showed that light scattering of material particles causes the Hb-encapsulated particles to have background slop^69^. A similar pattern of peak shifts for oxygenated Hb and deoxygenated Hb of encapsulated Hb with PCL is shown in Fig. 5 showed the oxygen-carrying capacity of prepared samples that is quite similar to free bovine-Hb^57,70^. The results reveal that the functionality, chemical structure, and oxygen binding and releasing ability of Hb were highly preserved. These results explicitly open the gate for the medical application of prepared particles.

The current study explored the antioxidant property, analysed by DPPH assay of developed particles. The antioxidant potential of these particles is still not reported. The colour change scheme measures the relative scavenging antioxidant efficiencies^71^. According to the studies reported by Jadid et al. if the IC50 has the range of (10–50 μg/mL) then the material has been considered a strong antioxidant agent as ascorbic acid^72,73^. Liu et al. previously reported that the polymer-based particles represented the strong antioxidant potential regarding the stability of particles and development of reactive oxygen species^74^. It was found that PCL itself has special antioxidant implications^75^. Free radicals keep a balance between the production and elimination process in the body for the life of cells. So previous studies reported by Wang et al. showed that haemoglobin based oxygen carriers (HBCOs) are used with excellent antiradical activities in many practical applications^76^. It is found in literature that for autoxidation of RBCs, within the cell, the non-functional metHb is reduced back into Hb by enzymatic and non-enzymatic control of antioxidant systems^77^. After some time, the Hb ultimately auto-oxidized into metHb due to Fe^2+^ iron to bind and losing oxygen. When Fe^3+^ oxidized, then the metHb showed no oxygen-carrying capacity. That’s why experiments show that the encapsulation of Hb with materials helps to reduce the formation of Hb to non-functional metHb^74,78^. It was found by Zare et al. that antiradical activity take place typically because of high surface-to-volume ratios of particle structures^79^. All the PCL-Hb particles have significant values referring to the positive control of free radical formation that may help to treat cancer cells and all other diseases caused by free radicals.

The developed PCL-Hb particles undergo experiments to check the bacterial growth (antibacterial agent) analysis that still has not been explored. It is reported in the literature that if some material has ZOI ≥ 6 mm, then it has antibacterial properties or vice versa. It has been reported previously by Hobson et al. that the haemoglobin’s activity against different bacteria still can’t be understood yet^80^. Moreover, bovine-Hb also revealed strong antibacterial properties and is an attractive source of antibacterial peptides^31,81^. Polymers also have strong antibacterial properties when used with antibacterial standard medicines for drug delivery systems. That may be related to the partial positive charge on (H-functional group) of PCL that breaks the wall membrane of bacterial cells and interacts^75,82^. Myriam et al. reported that PCL molecule has a large surface area to interact, so the composites developed by electrospun or 3-D printing techniques show enhanced antibacterial potential against *S.aureus* and *Staphylococcus epidermidis*^83^. Here we use polyvinyl alcohol (PVA) as emulsifying agent and the presence of (^–^OH) group made him an excellent antibacterial agent^84^. That ensures the addition of polymer increases the antibacterial property. Several reports showed that an increase in concentration of particles causes increase in the surface interaction of particles with bacteria walls^80,85^. That’s why the PCL-Hb particles have larger molecules that increase the surface-to-volume ratio to make them suitable for an antibacterial agent.

Furthermore, the MTT assay assessed the cell viability of synthesized PCL-Hb-based particles on animal fibroblasts cultured cell lines. For our goal, the increase in concentration doesn’t much alter the cell viability compared to the reference. That may be due to the cell recognition site, which will be provided by Hb-protein, thus resulting in high proliferation and cell adhesion. It was reported previously by Chen, Merkel et al. about minimal cytotoxicity at lower doses of reduced Hb-RBCM particles. However, as the particle dose increases, the cell viability declines, possibly due to an elevated formation of metHb within the cell culture^50,86^. It was reported previously by Selvaraj et al. that the PCL-based nano-fibres surface has shown supplementary protecting nature, which may enhance fibroblast proliferation and the significant (*P* < 0.05) increased the cell viability when PCL-based composites were used as antibacterial agents^87^. Their biocompatibility would rely on methHb formation level in particles within cell lines. We initiated to explore further reactive oxygen species (ROS) rummaging enzymes to resolve the dependence for upcoming future applications^71,87^. The results graphically shown in Fig. 9 concluded that the cells proliferated well, and the synthesized particles are nontoxic and cyto-compatible (biocompatible) in large concentration ranges. This Non-toxic potential promises the particles to be safe and biocompatible for many biomedical applications such as, artificial oxygen carriers, anti-cancer, and wound healing agents.

The stability and perseverance of both biological and chemical structure of encapsulated haemoglobin, Nano size of particles, non-toxic (biocompatible) nature, with enhanced oxygen carrying capacity, and P_50_ resulted from ODC and UV–visible, reversible oxygen affinity, comparable antioxidant properties, and novel antibacterial analysis made them excellent reference particles for artificial oxygen carrier, antibacterial and antioxidant agent as well as may be used for many other biomedical implications.

## Conclusions

We developed the polymer (PCL-Hb) based nanoparticles that functionalized the haemoglobin as encapsulating media. The biocompatibility and biodegradability of PCL made particles a potential source of multiple applications in biomedical physics. SEM analysis revealed that the particles are nano-sized with a spherical and porous structure that enhanced the oxygen affinity to serve as an artificial oxygen carrier. FTIR spectroscopy indicated a well-preserved chemical structure of amide-I (1600–1700 cm^−1^) and amide-II (1525–1575 cm^−1^) functional groups. The oxygen saturation profile observed by NIR-UV visible spectra and P_50_ values are measured. The ODC analysis showed that the prepared particles have nearly similar oxygen profiles and P_50_ values to bovine-Hb, which explored the functionality of nanoparticles. The UV–visible analysis referred to the maximum absorbance as the soret band (oxygenation), and effective shifts of the Q-band (de-oxygenation) indicated the well-preserved structure and functionality of oxygen binding in particles. However, the antioxidant potential of PCL-Hb nanoparticles, verified through the DPPH assay, has not been reported yet. The antiradical properties of these particles could prove beneficial in treating diseases induced by free radicals and cancerous cells. Similarly, the antibacterial property, determined using the disk diffusion method, explored the control of developed PCL-Hb particles on bacterial growth, particularly in the case of gram-negative bacteria compared to gram-positive bacteria. The biocompatibility (non-toxic nature) of PCL-Hb-based particles, confirmed by the MTT assay, along with the preserved structure and maintained functionality of Hb, makes these particles suitable for further in-vivo investigations in biomedical applications such as therapeutics, drug carriers, and contrast agents.

### Supplementary Information

Supplementary Information.

## Data Availability

The datasets used and/or analysed during the current study are available from the corresponding author on reasonable request.
