# Supplementary material for: Development of novel polymer haemoglobin based particles as an antioxidant, antibacterial and an oxygen carrier agents
Source: Sci Rep. 2024 Feb 6;14:3031. doi: 10.1038/s41598-024-53548-5 (PMC10847508; doi:10.1038/s41598-024-53548-5)
Supplement: Supplementary file 1 — Supplementary Information. [file 41598_2024_53548_MOESM1_ESM.docx]

# Development of Novel Polymer Haemoglobin based Particles as an Antioxidant, Antibacterial and an Oxygen Carrier Agents

# Antibacterial Activity of PCL-HB based particles

# Supplementary Table 1: **Zone of inhibition for different bacterial strains using PCL-Hb particles and control media**

| Sr # | Sample Name | Concentration Used | Name & Nature of Bacteria | ZOI Standard  Ave. Dia.(mm) | Standard Name | ZOI PCL/Hb  Ave. Dia.(mm) |
| --- | --- | --- | --- | --- | --- | --- |
| 1 | PCL-Hb_1%PVA_ (S1) | M1-10μL | *Pseudomonas*(SD) Gram-(-ve) | 19 | Amican | 15 |
|  |  | M2-20μL |  |  |  | 15 |
|  |  | M3-30μL |  |  |  | 21 |
| 2 |  | M1-10μL | *Bacillus* *cereus* ( BE) Gram-(+ve) | 23 | Amican | 9 |
|  |  | M2-20μL |  |  |  | 8 |
|  |  | M3-30μL |  |  |  | 23 |
| 3 |  | M1-10μL | *Staphylococcus* *aureus* (ST) Gram-(+ve) | 27 | Amican | 10 |
|  |  | M2-20μL |  |  |  | 26 |
|  |  | M3-30μL |  |  |  | 8 |
| 4 |  | M1-10μL | *E*.*coli* (EC)  Gram-(-ve) | 22 | Amican | 21 |
|  |  | M2-20μL |  |  |  | 20 |
|  |  | M3-30μL |  |  |  | 27 |
| 1 | PCL-Hb_5%PVA_ (S5) | M1-10μL | *Pseudomonas*(SD) Gram-(-ve) | 19 | Amican | 17 |
|  |  | M2-20μL |  |  |  | 19 |
|  |  | M3-30μL |  |  |  | 13 |
| 2 |  | M1-10μL | *Bacillus* *cereus* (BE)  Gram-(+ve) | 28 | Amican | 18 |
|  |  | M2-20μL |  |  |  | 30 |
|  |  | M3-30μL |  |  |  | 24 |
| 3 |  | M1-10μL | *Staphylococcus* *aureus* (ST) Gram-(+ve) | 15 | Amican | 27 |
|  |  | M2-20μL |  |  |  | 25 |
|  |  | M3-30μL |  |  |  | 8 |
| 4 |  | M1-10μL | *E.coli* (EC)  Gram-(-ve) | 17 | Amican | 20 |
|  |  | M2-20μL |  |  |  | 15 |
|  |  | M3-30μL |  |  |  | 16 |
